# Emergence of dual and multicarbapenemase coproducing organisms in the United States

**DOI:** 10.1017/ash.2022.6

**Published:** 2022-02-03

**Authors:** Bekana K. Tadese, Tolulope Olumuyiwa, Charles Darkoh

**Affiliations:** 1 Center for Infectious Diseases, Human Genetics, and Environmental Sciences, Department of Epidemiology, School of Public Health, University of Texas Health Science Center, Houston, Texas; 2 Fort Bend County Health and Human Services, Texas; 3 MD Anderson Cancer Center UTHealth Graduate School of Biomedical Sciences, Microbiology, and Infectious Diseases Program, Houston, Texas; 4 Houston Health Department, Houston, Texas

## Abstract

Carbapenemase-producing organisms commonly carry a single carbapenemase gene conferring resistant to carbapenems and other β-lactam antibiotics. Here, we report rare cases of multidrug-resistant *Pseudomonas aeruginosa*, *Klebsiella pneumoniae,* and *Acinetobacter baumannii* strains that coproduce multiple carbapenemases and exhibit extensive drug resistance. Such resistant strains are rarely identified in the United States.

The global spread of carbapenemase-producing organisms (CPOs) is an urgent public health threat due to lack effective drugs.^1,2^ Carbapenemase-producing bacterial pathogens are difficult to treat and are associated with increased severity of illness and mortality.^3^ The genes encoding carbapenemases are mostly located on mobile genetic elements that could be transferred horizontally to other gram-negative bacteria.^2,4^ In the United States, *Klebsiella pneumoniae* carbapenemase (KPC) is the most commonly identified carbapenemase among the carbapenem-resistant Enterobacterales (CRE).^3^ Oxacillinases (OXA) and other metallo-β-lactamases, such as New Delhi metallo-β-lactamase (NDM), verona integron-encoded metallo-β-lactamase (VIM), and imipenamase-producing metallo-β-lactamase (IMP), are not common and are particularly limited to healthcare-associated common-source outbreaks.^3,5^ NDM and OXA-48 are widespread in southeast Asia, Europe, China, and Africa.^3,6,8^ However, carbapenemase-producing carbapenem-resistant *Pseudomonas aeruginosa* (CP-CRPA) strains are reported rarely in North America, in part because medical laboratories do not routinely test for them.

Although CPOs usually carry single carbapenemase genes, few studies have reported dual or multicarbapenemase coproducing organisms.^7^ In the United States, the first case of *K. pneumoniae* coproducing NDM-1 and OXA-232 was reported in Pittsburgh.^9^ However, *P. aeruginosa* and *A. baumannii* coproducing different carbapenemases has never been reported. Here, we describe an unusual occurrence of patients infected with *K. pneumoniae, P. aeruginosa,* and carbapenem-resistant *A. baumannii* (CRAB) coproducing multiple carbapenemases.

## Methods

In Texas, clinical isolates identified to be resistant to multiple antibiotics at healthcare facilities are routinely referred to the Houston Health Department (HHD) and Antibiotic Resistant Laboratory network (ARLN) for further analysis. We examined these data for infections caused by different carbapenemase coproducing pathogens from 2018 to 2021. This research followed the first identification of a cluster of KPC-producing CRPA in the greater Houston area and their unusual antimicrobial susceptibility profiles. Bacterial isolates were identified by matrix-assisted laser desorption/ionization time-of-flight mass spectroscopy (MALDI-TOF MS). Confirmation of carbapenemase production was done using the modified carbapenem inactivation method (mCIM). Polymerase chain reaction (PCR) and whole-genome sequencing were used to identify carbapenemase genes: *bla*KPC, *bla*NDM, *bla*OXA-48-like, *bla*IMP, and *bla*VIM. Antibiotic susceptibility tests were performed using the broth microdilution method. Isolates showing rare resistance mechanisms were also sent to the Centers for Disease Control and Prevention (CDC) for further analysis. Patient demographics, microbiological, and clinical information were abstracted from the surveillance data and medical records. This study was reviewed and approved by the Institutional Review Board of University of Texas Health Science Center at Houston.

## Results

From January 2018 to July 2021, 7 patients infected with multidrug-resistant organisms coproducing multiple carbapenemases were identified. Table 1 shows the demographic, clinical, and other characteristics of the patients. Moreover, 4 patients were infected with CRE *K. pneumoniae,* 4 were identified with CRPA, and 1 was infected with CRAB. All patients were reported from different healthcare facilities and 5 patients had invasive renal devices (Table 1B).

Patient 1 had traveled to India and was hospitalized there for renal disease. The treatment in India was unsuccessful, and this patient was admitted to an acute-care hospital in Houston 4 days after arrival in the United States. A urine culture showed NDM and OXA-48 coproducing *K. pneumoniae* and NDM and IMP coproducing *P. aeruginosa.* The isolates were resistant to multiple antibiotics, including colistin and polymyxin (Table 2).

Patient 2 had a nephrostomy tube for ureteral stricture and hydronephrosis in Nigeria and was admitted to an acute-care hospital 1 week after arrival in the United States. Initial urine culture identified a carbapenemase-positive *P. aeruginosa*. Whole-genome sequencing, mCIM, and real-time PCR confirmed the presence of VIM and NDM carbapenemases. Antibiotic susceptibility test showed resistance to carbapenems, amikacin, tobramycin, gentamycin, and zerbaxa, a ceftolozane-tazobactam combination.

Patient 3 was admitted to the hospital for a cardiac problem. Urine culture identified *K. pneumoniae* with KPC and OXA-48 genes. Details of this patient’s medical history and healthcare abroad were not available.

Patient 4 had no history of travel abroad and was initially admitted to an intensive care unit. Wound culture revealed carbapenemase-producing *K. pneumoniae* harboring both KPC and VIM genes. In addition, a CRPA isolate harboring the VIM gene was also identified. The CRPA-VIM isolate was resistant to all antibiotics tested at the hospital. The patient was readmitted to an acute-care hospital 2 months later, and another urine culture also grew a highly resistant CRPA coproducing a VIM carbapenemase.

Patient 5 was a resident of a rehabilitation facility. CRPA was initially identified from routine urine culture, which was confirmed coproducing VIM and IMP carbapenemases. The patient’s medical history and healthcare abroad was not available.

Patient 6 had liver transplant and end-stage renal disease with a trialysis catheter port but developed intrabdominal complications. The patient had sepsis with *K. pneumoniae* bacteremia, which was identified to be coproducing NDM and KPC carbapenemases. Furthermore, a *bla*NDM-1 producing *E. coli* was also identified.

Patient 7 received outpatient foot wound care and culture from the wound grew carbapenem-resistant *A. baumannii*. Whole-genome sequencing and mCIM showed the presence of 3 carbapenemases: NDM, OXA-565 (OXA-23 like), and OXA-58. The patient had no recent history of medical care abroad, healthcare facility admission, or invasive medical device.

## Discussion

We describe a rare occurrence of multidrug-resistant organisms coproducing multiple carbapenemases in the United States, posing an urgent public health threat. These were *K. pneumoniae* coproducing KPC and OXA-48, NDM and OXA-48, KPC and VIM, KPC and NDM; *P. aeruginosa* coproducing NDM and IMP, NDM and VIM, IMP and VIM; and *A. baumannii* coproducing NDM and OXA variants OXA-565 (OXA-23-like) and OXA-58. Patient 1 was coinfected with *K. pneumoniae* coproducing NDM and OXA-48, and *P. aeruginosa* co-producing NDM and IMP carbapenemases. Although carbapenemase-producing *P. aeruginosa* infections are generally rare in the United States, the occurrence of 3 different carbapenemases in a single patient is unusual. Moreover, OXA-48 and NDM carbapenemases are also considered rare in the United States and their emergence here is alarming.

Two patients received invasive medical care in India and Nigeria, which may suggest potential importation of the infection. Although, the patients had 1 or more comorbidities, most of the cases had invasive medical procedures or devices, organ transplant, and surgery or wound care.

The *P. aeruginosa* and *K. pneumoniae* isolates identified to be coproducing different carbapenemases exhibited high resistance to aminoglycosides, including amikacin, tobramycin, and gentamicin, in addition to carbapenems. These findings are in line with previous reports of *K. pneumoniae* coproducing NDM-1 and OXA-232 and exhibited high level of resistance to amikacin and gentamycin.^9^

The *P. aeruginosa* isolate from patient 4 identified with blaVIM was resistant to multiple combinatorial drug therapies, including vabomere (meropenem and vaborbactam), avycaz (ceftazidime and avibactam), and zerbaxa (ceftolozane and tazobactam). These therapies are novel treatment strategies for complicated gram-negative infections. Moreover, the *P. aeruginosa* isolate from patient 1 coproducing IMP and NDM carbapenemases also exhibited resistance to all antibiotics tested, including colistin and polymyxin B, which are considered last-line drugs for extensively drug-resistant gram-negative infections.^9^ Although CRAB expressing OXA-23 like and OXA-40 has been previously reported in the United States,^10^ coproduction of multiple variants of OXA-565 (OXA-23-like) and OXA-58 with NDM has never been reported.

Notably, most hospitals and clinical laboratories might not be able to send isolates to public health laboratories for further analysis. Also, not all hospitals may have reported all cases to the health department. Therefore, our data may represent the tip of iceberg.

## Figures and Tables

**Table 1.** Clinical Risk Factors and Presumed Sources of Infection for the Carbapenemase-Coproducing Organisms

Patient	Underlying Comorbidities	Surgery or Wound	Invasive Medical Devices	Organ Transplant	Travel and Medical Care Abroad^a^
1	CKD with HD	Renal	Nephrostomy tube	Renal transplant	India
2	CKD with HD	Renal	Nephrostomy tube	No	Nigeria
3	CVD	Unknown	Unknown	Unknown	Unknown
4	Respiratory failure, renal failure	Sacral wound	Endotracheal tubes, Foley catheter	No	No
5	Unknown	Unknown	Unknown	Unknown	Unknown
6	ESRD with HD	Intrabdominal surgery	Intrabdominal stent	Liver transplant	No
7	Unknown	Foot wound	No	No	No

Note. MDRO, multidrug-resistant organism; CKD, chronic kidney disease; HD, hemodialysis; ESRD, end-stage renal disease; CVD, cardiovascular disease; mCIM+, carbapenemase positive by modified carbapenem inactivation method.

a Both case 1 and 2 had an invasive medical procedure abroad in the past month prior to the specimen collection.

**Table 2.** Antimicrobial Susceptibility Profiles of Carbapenemase Coproducing Organisms Isolated from the Patients

Variable	Case 1a	Case 1b	Case 2	Case 4a	Case 4b	Case 5	Case 6	Case 7
Isolate	KP	PA	PA	KP	PA	PA	KP	AB
Antibiotics	NDM & OXA-48	NDM & IMP	NDM & VIM	KPC &VIM	VIM	VIM & IMP	KPC &NDM	NDM and OXA-565 (OXA-23-like) and OXA-58
Amikacin	R	R	R	R	R	R	I	S^a^
Amoxicillin-clavulanate	R	R			R			
Ampicillin	R	R		R	R		R	
Aztreonam	R	R		R	R	R		
Ampicillin-sulbactam	R	R	R	R	R		R	R
Cefazolin	R	R		R	R			
Cefepime	R	R	R	R	R	R	R	R
Cefotaxime	R	R		R	R			S
Ceftazidime	R	R	R	R	R	R		R
Ceftriaxone	R	R		R	R		R	R
Cefuroxime	R	R		R	R			
Ciprofloxacin	R	R	R	R	R	R	R	R
Gentamicin	R	R	R	S	R	R	R	S
Ertapenem	R	R	R	R	R			
Imipenem	R	R	R		R	R		S
Levofloxacin	R	R	R	R	R	R	R	S
Meropenem	R	R	R	R	R	R	R	R
Doripenem	R	R	R	R	R			
Nitrofurantoin	R	R		R	R			
Piperacillin-tazobactam	R	R	R	R	R	S	R	R
Tetracycline	R	R		S	R		S	
Tigecycline	R	R			R			
Tobramycin	R	R	R	R	R	R		S
Piperacillin		R		R	R	R		
Trimethoprim-sulfamethoxazole	R	R		R	R		R	R
Ticarcillin	R	R			R	R		
Ticarcillin-clavulanate	R	R			R			R
Minocycline								S
Docycycline								S
Avycaz (ceftazidime/avibactam)					R		S	
Zerbaxa (ceftolozane-tazobactam)			R		R			
Vabomere (meropen-vaborbactam)					R			
Colistin, Polymyxin B		R			S		S	I

Note. KP, *K. pneumoniae*; PA, *P. aeruginosa*; AB, *A. baumannii*; R, resistant; I, intermediate, S, susceptible.

a Susceptibility report at clinical laboratories showed resistance to Amikacin. For the case 3, complete antibiotic susceptibility profile was not recorded in the surveillance and hence not included in this table.

## References

[1] Bonomo RA, Burd EM, Conly J, Carbapenemase-producing organisms: a global scourge. Clin Infect Dis 2018; 66: 1290–1297. 29165604 10.1093/cid/cix893 PMC5884739

[2] Queenan AM, Bush K. Carbapenemases: the versatile beta-lactamases. Clin Microbiol Rev 2007; 20: 440–458. 17630334 10.1128/CMR.00001-07 PMC1932750

[3] Van Duin D, Doi Y. The global epidemiology of carbapenemase-producing Enterobacteriaceae. Virulence 2017; 8: 460–469. 27593176 10.1080/21505594.2016.1222343 PMC5477705

[4] Martínez-Martínez L, González-López JJ. Carbapenemases in Enterobacteriaceae: types and molecular epidemiology. Enferm Infec Microbiol Clin 2014;32 suppl 4:4–9. 10.1016/S0213-005X(14)70168-5 25542046

[5] CDC. Notes from the field: New Delhi metallo-β-lactamase-producing Escherichia coli associated with endoscopic retrograde cholangiopancreatography—Illinois. 2013 Morb Mortal Wkly Rep 2014;62:1051. PMC4663693 24381080

[6] Avolio M, Vignaroli C, Crapis M, Camporese A. Coproduction of NDM-1 and OXA-232 by ST16 *Klebsiella pneumoniae*, Italy. 2016 Future Microbiol 2017; 12: 1119–1122. 28876082 10.2217/fmb-2017-0041

[7] Han R, Shi Q, Wu S, Dissemination of carbapenemases (KPC, NDM, OXA-48, IMP, and VIM) among carbapenem-resistant Enterobacteriaceae isolated from adult and children patients in China. Front Cell Infect Microbiol 2020;10:314. 32719751 10.3389/fcimb.2020.00314 PMC7347961

[8] Doi Y, O’Hara JA, Lando JF, Coproduction of NDM-1 and OXA-232 by *Klebsiella pneumoniae*. Emerg Infect Dis 2014; 20: 163–165. 24377764 10.3201/eid2001.130904 PMC3884727

[9] Cai Y, Lee W, Kwa AL. Polymyxin B versus colistin: an update. Expert Rev Anti Infect Ther 2015; 13: 1481–1497. 26488563 10.1586/14787210.2015.1093933

[10] Koirala J, Tyagi I, Guntupalli L, OXA-23– and OXA-40–producing carbapenem-resistant Acinetobacter baumannii in central Illinois. Diagn Microbiol Infect Dis 2020;97:114999. 32059871 10.1016/j.diagmicrobio.2020.114999

